# Comparative Efficacy of Hypoglossal Nerve Stimulation and Continuous Positive Airway Pressure in Obstructive Sleep Apnea: A Systematic Review

**DOI:** 10.7759/cureus.109549

**Published:** 2026-05-24

**Authors:** Eric Nguyen, Tomas Sepulveda

**Affiliations:** 1 Anatomy, Saba University School of Medicine, The Bottom, BES

**Keywords:** continuous positive airway pressure (cpap), cpap, electric stimulation, hypoglossal nerve, hypoglossal nerve stimulation, sleep apnea obstructive

## Abstract

This systematic review aims to compare the efficacy of hypoglossal nerve stimulation (HNS) versus continuous positive airway pressure (CPAP) for patients with obstructive sleep apnea (OSA).

A literature search was performed in PubMed and the Cochrane Library using keywords related to HNS, CPAP, and OSA, with a 10-year time limit (2014-2024). Five comparative clinical studies (four retrospective cohort studies and one prospective cohort study) were included. Outcome measures included the apnea-hypopnea index (AHI), mean disease alleviation (MDA), and Epworth Sleepiness Scale (ESS).

Both HNS and CPAP significantly reduced AHI from baseline in all included studies (p < 0.001). Between-group differences in AHI reduction were inconsistent across studies. MDA was higher in HNS patients than in CPAP patients in two studies (59% vs. 51%; 22.9% vs. 5.0%). All studies demonstrated statistically significant improvements in ESS scores in both groups; most studies favored HNS, while one study showed a slightly greater improvement with CPAP.

Both treatments effectively reduce respiratory events in OSA. Due to high heterogeneity, variable follow-up periods, and non-randomized designs, no definitive conclusion can be drawn that HNS is superior to CPAP. However, HNS is associated with better patient-reported outcomes and higher real-world effectiveness, likely related to superior adherence. HNS represents a valuable alternative for patients who are intolerant to CPAP. Future large-scale randomized controlled trials are required to confirm comparative efficacy.

## Introduction and background

Obstructive sleep apnea (OSA) is a highly prevalent chronic sleep-related breathing disorder characterized by recurrent partial or complete upper airway collapse during sleep, leading to intermittent hypoxia, sleep fragmentation, and daytime somnolence [1]. Globally, OSA affects approximately one billion individuals, with 425 million people living with moderate-to-severe disease [1]. The rising prevalence is strongly associated with the global obesity epidemic, with body mass index (BMI) being the most significant modifiable risk factor [2]. Non-modifiable risk factors include advanced age, male sex, increased neck circumference, and craniofacial anatomical abnormalities [1]. The male-to-female prevalence ratio is approximately 2:1 worldwide [3]. Untreated OSA is linked to severe cardiovascular comorbidities, including hypertension, atrial fibrillation, heart failure, and cerebrovascular events, representing a major global public health burden [1].

Continuous positive airway pressure (CPAP) is the current standard of care for treating OSA and has demonstrated high efficacy in maintaining the upper respiratory tract patency, thereby preventing apnea episodes [3]. However, the main limitation of CPAP is patient adherence, with non-adherence defined as use of less than four hours per night on 70% of nights' sleep [1]. About 14.9%-35.5% of patients cannot tolerate CPAP, and as a result, many patients are undertreated with CPAP, which leads to persistent symptoms and/or cardiovascular risks [3]. This limitation prompted the development of alternative therapeutic approaches, whether surgical or non-surgical, to help optimize patient adherence and outcomes [3].

**Figure 1:**
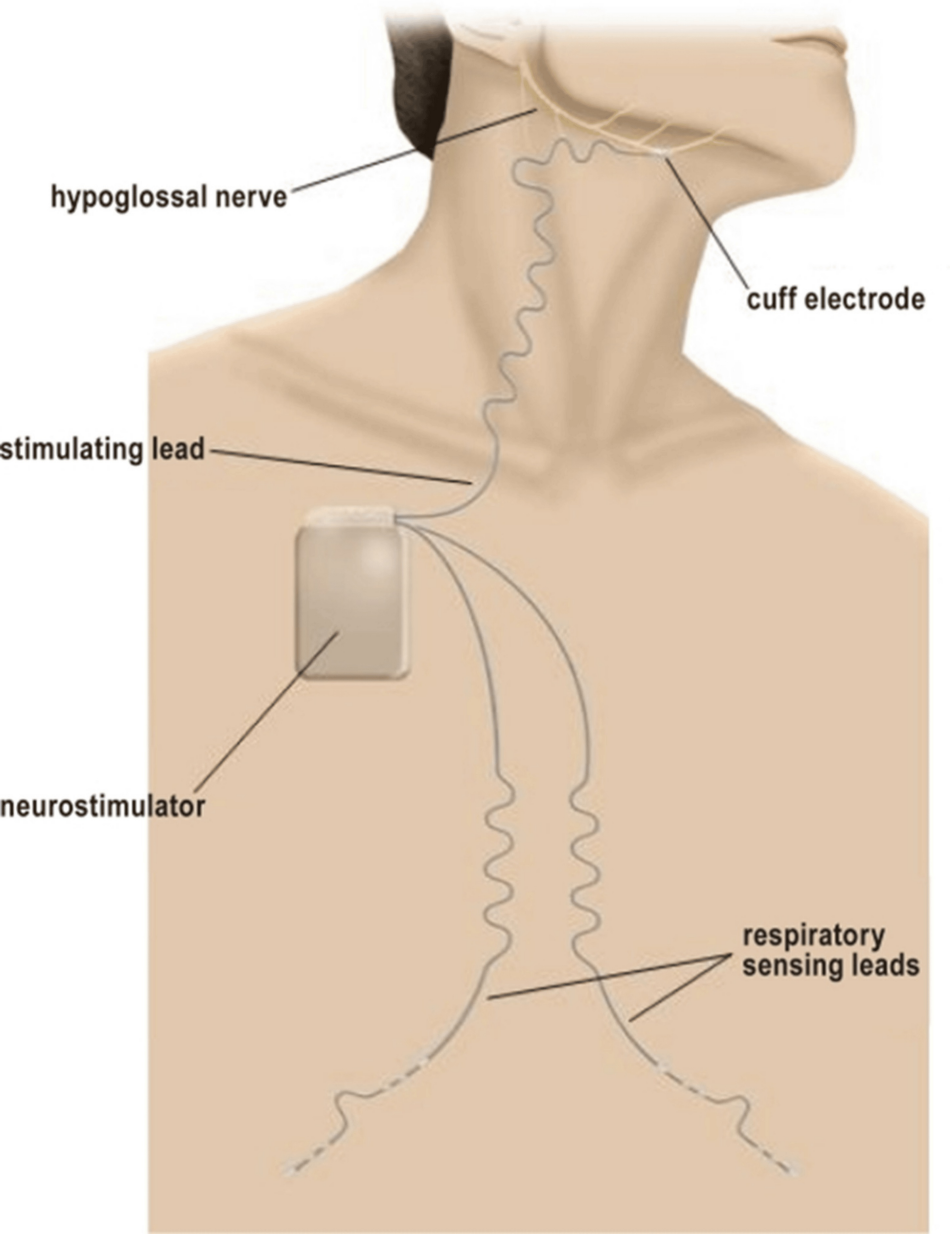
Anterior view of the hypoglossal nerve stimulation device. Chest pulse generator, a cuff electrode attached to the hypoglossal nerve, and an intercostal sensing lead for ventilatory effort. Reproduced from Eastwood PR, Barnes M, Walsh JH, et al., Treating obstructive sleep apnea with hypoglossal nerve stimulation, Sleep, 2011, 34:1479-1486 by permission of Oxford University Press [5].

**Figure 2:**
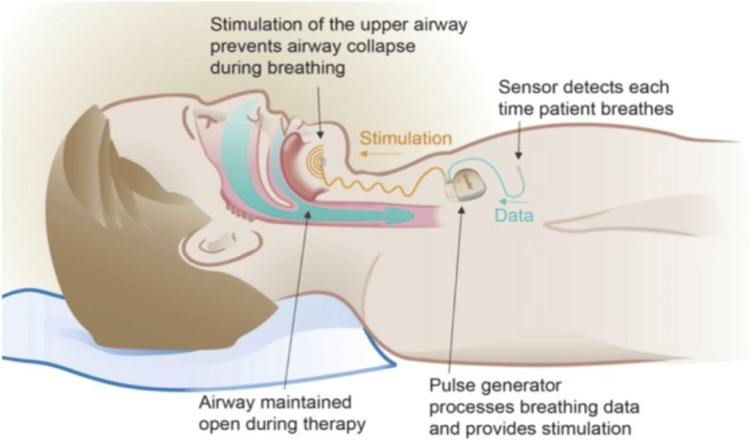
Implantable hypoglossal nerve stimulation system with three components: (1) chest pulse generator, (2) a cuff electrode attached to the hypoglossal nerve, and (3) an intercostal sensing lead for ventilatory effort. Reproduced from Dedhia RC, Strollo PJ, Soose RJ, Upper airway stimulation for obstructive sleep apnea: past, present, and future, Sleep, 2015, 38:899-906 by permission of Oxford University Press [6].

**Figure 3:**
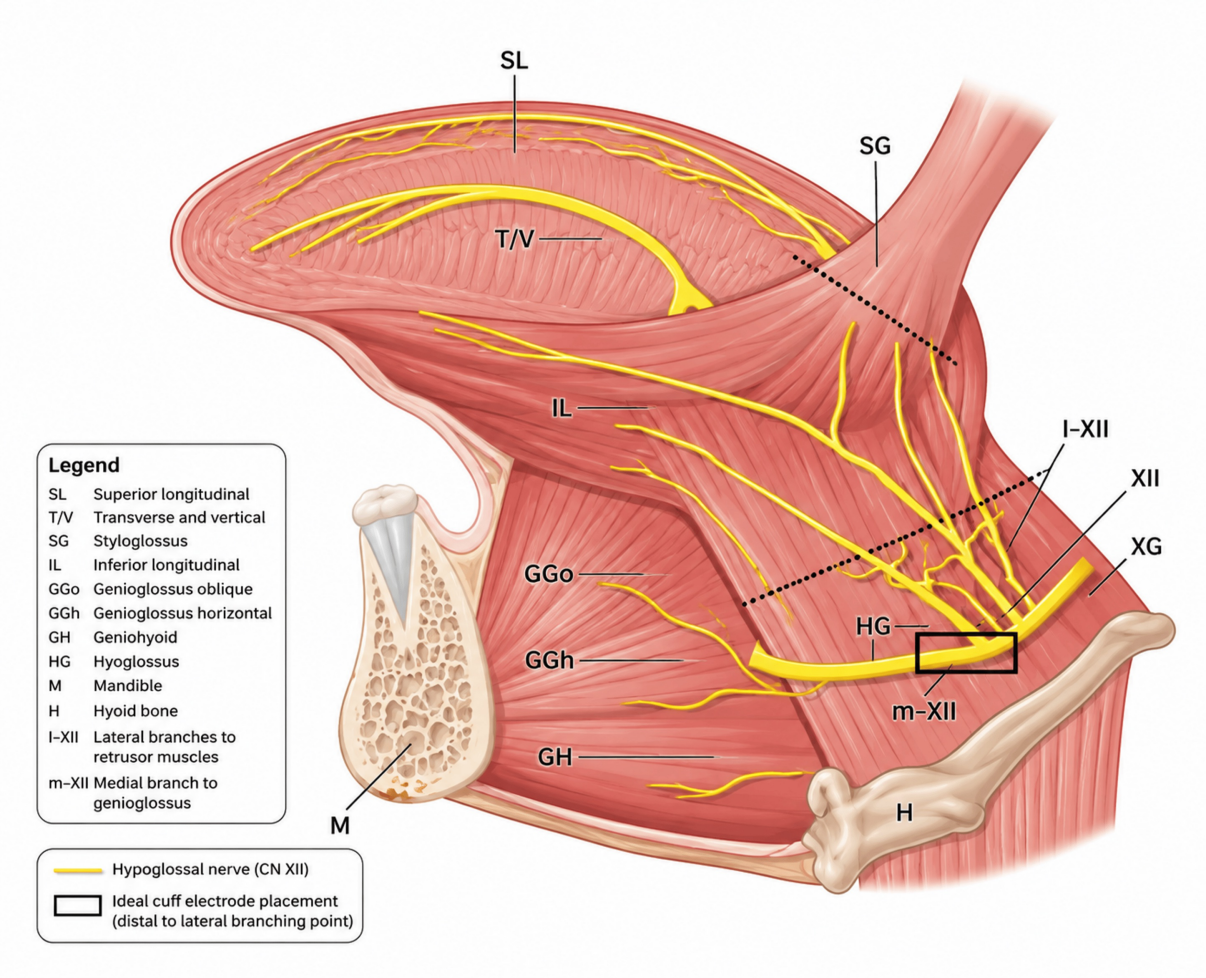
Neuroanatomy of the tongue. Sagittal view of tongue neuroanatomy showing hypoglossal nerve branches: lateral (styloglossus, hyoglossus) and medial (genioglossus). Ideal cuff electrode placement is distal to the lateral branching point. Reproduced from Dedhia RC, Strollo PJ, Soose RJ, Upper airway stimulation for obstructive sleep apnea: past, present, and future, Sleep, 2015, 38:899-906 by permission of Oxford University Press [6].

Hypoglossal nerve stimulation (HNS), also known as upper airway stimulation (UAS), is a surgical implant device approved by the FDA in 2014 [2], as seen in Figures 1-2. A type of HNS is Inspired (brand name), which works by synchronizing tongue protrusion with inspiration to maintain airway patency while sleeping [4]. The device is implanted in the right ipsilateral mid-infraclavicular region, similar to a pacemaker, and can be controlled remotely [4] as shown in Figures 1-2. It has a sensing lead on the intercostal muscles to detect respiratory effort, while stimulatory electrodes are attached to the protruding fibers of the right hypoglossal nerve (CN XII) [4] as shown in Figure 3. This is indicated for CPAP-intolerant patients who have a BMI < 35 kg/m² with moderate/severe OSA by improving objective sleep parameters, quality of life, and increasing adherence [4].

The objective of this systematic review is to evaluate the comparative efficacy of HNS and CPAP using primary (apnea-hypopnea index (AHI) and mean disease alleviation (MDA)) and secondary (Epworth Sleepiness Scale (ESS) and patient-reported outcomes (PROs)) variables to investigate whether HNS can potentially be a first-line treatment for OSA. Objective data, namely the AHI, are obtained via polysomnography (PSG) or at-home studies. AHI is a measure of OSA severity, and therapeutic efficiency is the change from baseline, which measures the degree of improvement [7]. As an extension of AHI, the concept of MDA was introduced into respiratory sleep medicine to evaluate the overall effectiveness of treatments for OSA [8]. MDA accounts for both adherence and therapeutic efficiency (Appendix A) [9]. ESS and PROs are used as secondary variables to evaluate the impact on quality of life in patients with OSA [8,10-13]. We hypothesized that HNS is a more effective treatment for OSA compared to CPAP.

## Review

Methods

This research was conducted on the search engines PubMed and Cochrane Library from 2014 to 2024. The search strategy included keywords relating to "hypoglossal nerve stimulation", "hypoglossal nerve", "electric stimulation", and/or "continuous positive airway pressure", "CPAP", and "Sleep apnea Obstructive". MeSH terms and Boolean operators (AND/OR) were used to increase search sensitivity. Variable interests, such as AHI, were initially excluded to increase sensitivity but were later added for specificity. References for articles of interest were carefully screened for similar studies.

Studies directly comparing the efficacy of HNS and CPAP in adults with mild to severe OSA were eligible for primary article analysis. At least one quantitative outcome must be reported, such as AHI, MDA, ESS, or PROs. Inclusion criteria included randomized controlled trials, comparative studies, and cohort studies. Exclusion criteria included case reports, narrative reviews, studies without quantitative data, and those outside of the specified 10-year period.

**Figure 4:**
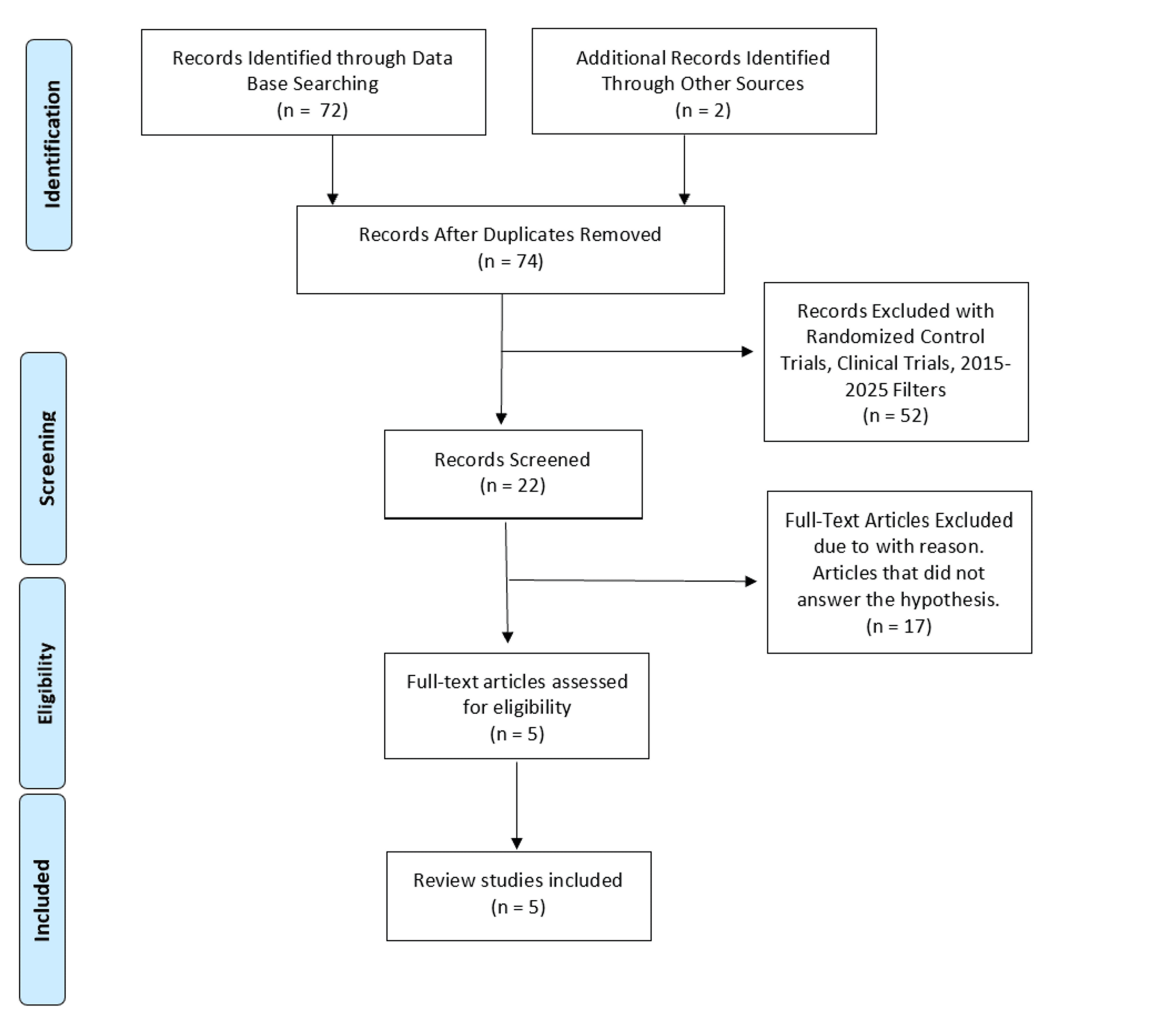
A PRISMA flowchart outlining the study selection process.

One reviewer extracted multiple studies’ characteristics and quantitative outcomes (AHI, MDA, ESS, and PROs) into a Microsoft Excel file (Microsoft Corp., Redmond, WA, USA). The Newcastle-Ottawa Scale was used to assess the cohort study for risk of bias. The validity was also considered when interpreting the data, and the decision to keep the article’s results was made. All variables of interest were compiled into comparative tables between studies. Manual calculation was done on therapeutic efficiency, MDA, and ESS change when data were sufficiently available. A meta-analysis could not be done because of the study heterogeneity. Figure 4 presents the PRISMA flowchart outlining the study selection process.

Results

This section presents the results of five studies to determine the efficacy of treatments between HNS (Figure 1 and Figure 2) and CPAP in treating OSA using the type of studies summarized in Table 1. First, we will compare the primary measures of efficacy using variables such as AHI and MDA before and after treatment. This will be followed by a comparison of the secondary measures of the studies, namely the ESS and PROs, to determine the relative impact of HNS and CPAP on patients' quality of life. 

**Table 1:** Types and number of studies selected for the comparison of primary and secondary measures of efficacy between hypoglossal nerve stimulation and continuous positive airway pressure.

Study designs	Number of studies
Retrospective cohort study	4
Prospective cohort study	1

Heiser et al. [10] compared HNS and CPAP in a prospective cohort study (n = 227) with 12-month follow-ups. Of the 227 patients who received treatment, 117 patients belonged to the UAS group, and 110 patients belonged to the PAP group. Baseline AHI was similar between the two groups (HNS: 36.5 ± 14.8 vs CPAP: 39.6 ± 26.7; p = 0.146), as shown in Table 2. Post treatment showed a statistically significant reduction in AHI in both groups (HNS: 8.1 ± 6.3/h; PAP: 6.6 ± 8.0/h; p < 0.001), as shown in Table 2, although absolute reduction showed no major differences. At follow-up, adherence was higher in the HNS than PAP therapy (5.0 ± 2.6 h/night vs. 4.0 ± 2.1 h/night); the difference was not statistically significant [10]. The MDA was 59% vs 51% for those receiving HNS and CPAP, respectively, as shown in Table 3.

**Table 2:** Comparison of AHI and MDA across studies. AHI: apnea–hypopnea index; ΔAHI: change from baseline; HNS: hypoglossal nerve stimulation; CPAP: continuous positive airway pressure; MDA: mean disease alleviation; NS: not significant

Studies	Treatment	Baseline AHI (mean ± SD)	Post-treatment AHI (mean ± SD)	Therapeutic efficiency (ΔAHI)	Within-group significance	Between-group difference	MDA (%)
Heiser [10]	HNS	36.5 ± 14.8	8.1 ± 6.3	28.4	p < 0.001	NS (p = 0.075)	59
	CPAP	39.6 ± 26.7	6.6 ± 8.0	33.0	p < 0.001		51
Pordzik [11]	HNS	40.2 ± 12.8	30.2 ± 17.7	8.0	p < 0.001	Favor CPAP (p< 0.001)	—
	CPAP	39.2 ± 12.3	4.7 ± 3.4	34.5	p < 0.001		—
Diecidue [9]	HNS	37.1 ± 15.7	10.1 ± 10.0	27.0	p < 0.001	Favor HNS (p = 0.004)	22.9 ± 13.2
	CPAP	32.6 ± 19.6	26.1 ± 22.6	6.5	p < 0.001		5.0 ± 14.8

**Table 3:** Calculating the MDA and overall remaining AHI for both treatments, HNS, and PAP. MDA: mean disease alleviation; HNS: hypoglossal nerve stimulation; PAP: positive airway pressure; AHI: apnea–hypopnea index Reference: [10]

	HNS cohort	PAP cohort
Therapeutic efficacy (%)	76	82
Adjusted adherence (%)	78	62
MDA (%)	59	51
Overall remaining AHI (/hour sleep)	14	18

For the secondary measures, the baseline ESS was statistically higher in the HNS group than the PAP group (15.4 ± 3.5 vs. 14.6 ± 3.9) [10], shown in Table 4. In other words, the HNS group had higher sleepiness symptoms reported relative to the PAP group at baseline. Post treatment, ESS showed a statistically significant improvement in the HNS group (HNS: 8.1 ± 6.3/h; PAP: 6.6 ± 8.0/h; p < 0.001) [10] as shown in Table 4.

**Table 4:** Comparison of ESS across studies; data synthesized by the authors from published studies. ESS: Epworth Sleepiness Scale; ΔESS: change from baseline; CI: confidence interval; HNS: hypoglossal nerve stimulation; CPAP: continuous positive airway pressure.

Study	Treatment	Baseline ESS (mean ± SD)	Post-treatment ESS (mean ± SD)	ΔESS	Within-group significance	Between-group difference
Heiser [10]	HNS	15.4 ± 3.5	8.1 ± 6.3	7.3	p < 0.001	−0.7 (favor CPAP)
	CPAP	14.6 ± 3.9	6.6 ± 8.0	8.0	p < 0.001	
Pordzik [11]	HNS	13.3 ± 5.8	7.2 ± 5.1	6.1	p < 0.001	5.4 (favor HNS)
	CPAP	9.1 ± 4.7	8.4 ± 5.4	0.7	—	
Pascoe [12]	HNS	11.2 (95% CI: 10.1–12.2)	7.9 (95% CI: 6.5–9.2)	3.3	—	—
Walia [13]	HNS	10.7 (95% CI: 9.8–11.6)	7.2 (95% CI: 6.4–8.1)	3.5	p < 0.001	0.8 (p = 0.046; favor HNS)
	CPAP	10.4 (95% CI: 9.5–11.2)	7.7 (95% CI: 6.8–8.6)	2.7	p < 0.001	
Diecidue [9]	HNS	10.9 ± 4.7	6.4 ± 4.4	4.4	p < 0.001	2.9 (favor HNS)
	CPAP	8.6 ± 5.1	7.1 ± 4.4	1.5	p < 0.001	

Pordzik et al. [11] compared the pre- and post-therapeutic AHI/ESS between HNS (n = 20) and automatic PAP (aPAP; n = 35) in a retrospective cohort study. Patients were selected based on standard OSA criteria. Baseline AHI and oxygen desaturation index (ODI) were comparable between the groups (HNS: 40.2, 37.9/h; aPAP: 39.2, 34.6/h), as detailed in Table 2. Follow-up occurred on average 413.6 days (hypoglossal nerve stimulation (HGNS)) and 162.09 days (aPAP) after treatment initiation. Post-therapeutic AHI remained significantly higher in the HGNS group compared to the aPAP group (HGNS: 30.2/h, aPAP group: 4.7/h; p < 0.001), as shown in Table 2. Post-interventional ESS was improved in both groups with a statistically significant reduction in the HNS group (HGNS: 13.32 to 7.2 points, aPAP: 9.09 to 8.38 points; p < 0.01), summarized in Table 4.

Pascoe et al. [12] conducted a retrospective cohort study at the Cleveland Clinic to evaluate PROs between HNS and PAP groups who shared similar demographic profiles. The HNS group achieved better ESS results than PAP patients since 64.6% of HNS patients (42 out of 65) showed improvement compared to 54.5% of PAP patients (118 out of 217). The HNS group demonstrated superior results compared to the PAP group across all three assessment tools: Functional Outcomes of Sleep Questionnaire (FOSQ; 59.2% vs 30.9%), Patient Health Questionnaire-9 (PHQ-9; 29.2% vs 24.4%), and Insomnia Severity Index (ISI; 46.9% vs 36.4%).

After adjusting for baseline variables (baseline PRO, age, sex, BMI, and AHI category), the HNS cohort had a 1.48-point greater decrease in PHQ-9 compared to the PAP cohort at the three-month follow-up (−4.06 (95% CI, −5.34 to −2.79) vs −2.58 (95% CI, −3.35 to −1.82); mean difference, −1.48 (95% CI, −2.78 to −0.19)). ESS, FOSQ, and ISI scores also showed a higher improvement from baseline at one, three, six, and 12-month follow-ups in the HNS cohort compared to the PAP cohort. At the 12-month follow-up for post-HNS patients, further improvement was seen in 17 of 28 patients (60.7%) for ESS scores, 11 of 20 patients (55.0%) for FOSQ scores, seven of 23 patients (30.4%) for PHQ-9 scores, and 11 of 25 patients (44.0%) for ISI scores.

Walia et al. [13] compared the effect of UAS (HNS) on ESS and blood pressure with PAP, providing insight into the impact of HNS on patients' quality of life. The retrospective cohort study consisted of 517 patients with OSA (AHI: 15-65; BMI < 35 kg/m^2^) who started on PAP therapy (2010-2017) and 320 patients with UAS implantation (2015-2017). Post-treatment PAP had a greater improvement in diastolic BP (mean difference, 3.7 mmHg; p < 0.001) and mean arterial pressure (MAP) (mean difference, 2.8 mmHg; P = 0.008) compared with UAS. However, UAS showed greater improvement in ESS scores vs PAP (mean difference -0.8; p = 0.046).

The cohort study by Diecidue et al. [9] evaluated four OSA treatment methods through a retrospective analysis of 119 patients who received CPAP, mandibular advancement device (MAD), UAS, or MMA from 2018 to 2020. The study population consisted mainly of white male patients who averaged 55 years old when using CPAP and 63.27 years old when using HNS. The study included 37 patients in the HNS group and 25 patients in the CPAP group, with a beta value of 0.39 across all four treatment groups. The initial AHI measurements showed no significant differences between the treatment groups (HNS: 37.1 ± 15.7; CPAP: 32.6 ± 19.6). The HNS group achieved a more significant reduction in mean AHI post-treatment (10.1 ± 10.0) than the CPAP group (26.1 ± 22.6), as shown in Table 2. The treatment efficacy percentage was calculated through pre- and post-AHI value differences, which showed higher results in the HNS group compared to CPAP (27% vs 6.4%, p = 0.004), as shown in Table 2. The results of adjusted compliance percentage showed no significant difference between the two groups (HNS: 85.3% ± 17.6; CPAP: 89.1% ± 18.0). The HNS group achieved the second-highest MDA value, which exceeded the CPAP group results (HNS: 22.9 ± 13.2; CPAP: 5.0 ± 14.8; p < 0.001), summarized in Table 2.

Secondary measures included ESS, Patient-Reported Apnea Questionnaire (PRAQ), and Sleep Apnea Quality of Life Index (SAQLI). Notably, post-PRAQ scores revealed a statistically significant improvement in quality of life with HNS compared to CPAP groups (Group score differences: t = −1.50, p = 0.001) and versus MAD and MMA as well (t = −1.71 and −1.80, both p < 0.001). In addition, ESS scores favored HNS, with baseline values that were higher in the HNS group (10.9 ± 4.7 vs 8.6 ± 5.1), with statistically significant improvement in post-treatment scores with HNS versus CPAP (HNS: 6.4 ± 4.4; CPAP: 7.1 ± 4.4; p = 0.03), as summarized in Table 4. Patients with improvement in MDA did not always align with survey measures; only ESS difference scores were significantly predictive of MDA in this study (t = −3.544, P < 0.0001), while the other surveys were not.

Discussion

The purpose of this article is to determine if HNS is associated with greater efficacy than CPAP for the treatment of OSA. Whether HNS can be considered a first-line treatment for OSA will be explored. The results will be analyzed using factors such as baseline and post-treatment AHI, MDA, ESS, and PROs to determine the implications of the significant difference in the data. Limitations and possible confounders will be examined to appraise the credibility of the studies' results. Future directions and areas requiring more exploration will be discussed.

This systematic review summarizes five comparative studies evaluating HNS versus CPAP for OSA. Both treatments significantly reduce AHI and improve daytime sleepiness. However, between-group efficacy results are inconsistent, and high heterogeneity prevents definitive conclusions.

AHI and Therapeutic Efficacy

All studies confirmed that both HNS and CPAP reduce AHI. However, results were conflicting: Heiser et al. found no significant difference between groups [10]. Pordzik et al. favored CPAP [11]. Diecidue et al. favored HNS [9]. These discrepancies likely reflect differences in sample size, follow-up duration, baseline disease severity, and adherence. CPAP provides stronger immediate airway patency, but HNS avoids adherence barriers of mask-based therapy.

Mean Disease Alleviation

MDA reflects real-world effectiveness by combining efficacy and adherence. Two studies showed higher MDA with HNS, likely driven by better long-term adherence [9, 10]. This supports the concept that HNS may be more effective in clinical practice, even if not more efficacious in controlled settings.

Daytime Sleepiness and Quality of Life

Most studies demonstrated significantly greater improvements in ESS and PROs with HNS, likely due to higher acceptance and adherence. Only Heiser et al. showed a slight advantage for CPAP, which was clinically negligible. The minimal clinically important difference (MCID) for ESS is ≥2 points; all studies exceeded this threshold for HNS.

Cardiovascular Effects

Walia et al. showed greater blood pressure reduction with CPAP, likely related to continuous positive pressure reducing intrathoracic pressure and sympathetic overactivity. This difference may be important for high-risk cardiovascular patients [13].

The changes in AHI between baseline and post-treatment vary by study, as shown in Table 3. Heiser et al. [10] did not find significant differences between HNS vs PAPs in terms of changes in AHI between the two groups (p = 0.075). Despite this, a significant difference was observed in both groups from baseline, which indicates both treatments can be effective in reducing AHI (p < 0.001). The group's baseline characteristic was homogeneous, including AHI, which suggests that this result minimized confounders and the sample size was sufficiently large to avoid a Type 2 error. The benefit of a prospective cohort study is that the researcher can control data collection in multiple hospitals and avoid confounders, as opposed to a retrospective cohort study. Limitations include a lack of randomization in this type of cohort study due to ethical concerns regarding the randomization of a first-line and a second-line treatment. As a result, patients' gender cannot be randomized, resulting in a higher proportion of females in the CPAP group, which does not reflect the real-world discrepancy. This limitation has a modest impact on external validity and is understandable given the study's design. The author notes baseline measurement of AHI was done in lab polysomnography, which could differ from the follow-up AHI measurement that was done at home, and thus may underestimate the OSA burden. However, given that two different measurements were completed at two different time points for all groups, this was not a significant factor in the outcome of the study. For future direction, the gender difference between groups can be addressed by selecting an equal or similar male-to-female ratio.

In contrast, Pordzik et al. [11] found that the post-treatment AHI was higher among the HNS group compared to the PAP group (p < 0.001). In other words, the authors' data suggest HNS is less effective at reducing the number of apnea episodes experienced by patients. The study's demographics more closely reflect the real world's 2:1 male-to-female ratio, but gender cannot be randomized, given that this study is a retrospective cohort study. These findings may suggest that PAP is more effective at reducing AHI (88%) compared to HNS (83%); however, due to the small sample size for both groups (HNS: n = 20; and PAP: n = 35), this finding should be interpreted with caution. The author argues that the post hoc power, calculated using a t-test and a two-sided significance level of alpha = 0.05, still shows the post-treatment AHI for PAP as statistically significant, which suggests that the study size is sufficient to prevent a type 2 error. In this case, the larger population PAP group (n=35) is enough to reduce the study beta to <20%. However, given the retrospective nature of the study, there is a possibility of observer bias or selection bias, particularly Berkson's bias, due to the subjects being sampled from a single hospital. Further evaluation of the patient sampling population, in conjunction with other quantitative measures, specifically MDA, is needed to understand the efficacy difference between HNS and CPAP treatments fully.

Diecidue et al. [9] found that AHI reduction was greater in the HNS cohort compared to the PAP cohort (p = 0.004; p < 0.05). This study is one of the first retrospective cohort studies to directly compare the efficacy of surgical vs. non-surgical treatment for OSA. The HNS group consisted of a larger HNS sample size (n = 37) with a similar gender ratio to the PAP population (n = 25). Due to the small sample size, this study reports a beta of 0.39 across the four groups, which exceeds the 20% threshold, indicating the possibility of a type 2 error. To help alleviate concerns about potential biases, the authors assess covariates, including age, sex, height, weight, socioeconomic status (SES), education level, neck size, race, and BMI, in relation to the measured outcomes of AHI, MDA, and PROs. Although pre-treatment statistical differences were present between each group, the author controlled for this and found that the main effects, namely the change in pre- and post-treatment AHI, were significant between the HNS and CPAP cohorts. Interestingly, an important limiting factor in this study may be race. The majority of the surgical treatment groups (MAD and HNS) consisted mainly of White patients compared to the non-surgical groups (MMA and CPAP). Race is a potential confounder, as specific demographics may have access to different insurance plans and healthcare services. It is possible that the result is due to White individuals responding better to surgical alternative treatment for OSA or that surgical intervention is superior in reducing AHI compared to non-surgical intervention. Thus, future studies should include a larger sample size and a careful treatment analysis based on race to understand if this relationship is impactful.

One of the primary quantitative measures and arguably the most impactful variable for assessing the efficacy of HNS and CPAP is the MDA. Comparing the MDA results of Heiser et al. [10] and Diecidue et al. [9], both studies show that HNS has a greater MDA than the CPAP cohort, as seen in Table 1. In Heiser et al. [10], patients' HNS adherence is approximately one hour longer than CPAP, which explains the difference in MDA, although the therapeutic efficacy remains comparable between the two groups. In conjunction with the adjusted compliance used and baseline variables matched between the two groups, these factors are unlikely to account for the observed differences. In Diecidue et al. [9], there was a greater reduction of AHI in the HNS group compared to the CPAP group. In contrast, Diecidue et al. [9] observed an approximately 4% higher adjusted compliance in the CPAP group compared to the HNS group (HNS adjusted compliance: 85.33% vs. CPAP: 89.14%). However, the treatment efficacy remains higher in the HNS group, thus explaining the higher MDA score in the HNS group. These study results suggest that the increase in MDA can be attributed to either greater adherence or better AHI improvement. Heiser et al. [10] noted that patients in the HNS group are typically those who cannot tolerate CPAP; thus, if they are more likely to experience subjective improvement with HNS, they are also more likely to remain adherent for a longer period. Although it is still unclear whether the respective treatments are dose-dependent, generally, patients who remain adherent to their treatment have a greater improvement in their OSA symptoms.

Of the five studies analyzed in Table 4 for baseline/post-treatment ESS, all results observed statistically significant changes in ESS with HNS treatment compared to CPAP. Pascoe et al. [12] found that after activating HNS for one month, ESS and other PRO variables improve significantly, with a 3.27 change from pre-ESS to post-ESS. Walia et al. [13] reported a similar improvement in the HNS group's ESS of 3.5 points, compared to a 2.7-point improvement in CPAP (difference: -0.8; p = 0.046). It is important to note that a larger decrease in ESS score from baseline to post treatment indicates a lower ESS score, which means that patients reported less daytime sleepiness. According to Pascoe et al. [12], the MCID for ESS for a patient with moderate to severe symptoms is a two-point difference. Both of these studies use a larger population size than the previous studies discussed and have sufficient power to determine this relationship. Pascoe et al. [13] found that their results did not account for HNS titration adjustments during the collection period. This is a possible confounder that may explain why patients with more HNS adjustments would have a greater improvement in PRO scores, or it may be due to the opposite. As mentioned previously, retrospective cohort studies are susceptible to missing data, which makes interpreting the results difficult. However, there is no difference in baseline and follow-up subject characteristics, and consistent improvement in ESS makes this finding negligible. This study can be improved by providing PROs change in the CPAP group consistent with the follow-up period seen in HNS for direct comparison. Walia et al. [13] were limited by possible systemic bias due to non-standardized measurement tools; however, this finding only applies to BP measurement, not ESS measurement.

Pordzik et al. [11] and Diecidue et al. [9] found a significant improvement in ESS when using HNS, with ESS changes of 6.15 and 4.44, respectively, compared to 0.71 and 1.54 in the CPAP group. This improvement further supports the notion that HNS is superior to CPAP in reducing ESS. This relationship is likely explained by the same reason as the change in MDA, meaning this difference may be due to patient adherence. HNS will remain activated once installed and is at a lower risk of non-adherence than CPAP. However, if this were the case, it would not explain why Heiser et al. [10], which had a higher patient adherence in the HNS group, was the only study where change in ESS was greater in the CPAP group, albeit only a slight difference (HNS ΔESS: 7.3; CPAP ΔESS: 8). This finding suggests another relationship other than adherence is at play, but it is more likely that this difference is negligible.

Pascoe et al. [12] investigated PROs to better understand the impact on quality of life and efficacy of HNS compared to CPAP. Aside from the ESS previously discussed, the FOSQ, ISI, and PHQ-9 were also investigated to understand the effect of comorbidities, which often accompany OSA, such as insomnia and depression. At three months, the clinical importance difference was seen in all groups treated with HNS. The FOSQ score in the HNS group was 59.2%, compared to 30.9% in the CPAP group, indicating that HNS reduces the effect of sleepiness on the ability to conduct daily activities more than CPAP. The improvement in PHQ-9 was seen in HNS compared to CPAP (29.2% vs 24.4%), which indicates HNS can reduce depression symptoms associated with OSA better than CPAP. Lastly, improvements in ISI were observed in HNS compared to CPAP (46.9% vs. 36.4%), suggesting that HNS is more effective in reducing the impact of insomnia symptoms. Subsequent follow-up showed similar findings. These findings suggest that HNS is a superior treatment option for improving the quality of life associated with OSA, thereby enhancing treatment efficacy. One limitation of this study was that the mean follow-up time between the two groups differed; thus, future directions could include a strict protocol for follow-up visits to minimize potential confounding.

To comprehensively assess the efficacy of HNS compared to CPAP, it is also necessary to discuss other effects on comorbidities related to OSA. Walia et al. [13] further support HNS showing a greater improvement in ESS scores compared to PAP (mean change = 0.8; p = 0.046). This magnitude of change was again due to greater adherence in the HNS group. However, the impact of CPAP on blood pressure in this study, compared to HNS, suggests otherwise. The CPAP cohort showed a statistically significant reduction in diastolic BP (mean = 0.7 mm Hg; P < .001) and MAP (mean = 2.8 mm Hg; P = .008) [13]. The authors propose that this is due to the direct mechanical influences through positive pressure when using CPAP and its ability to reduce intrathoracic pressure swings. In other words, although both treatments can improve hypoxemia, which is a key driver for hypertension via endothelin release and increased vascular smooth muscle tone, CPAP additionally relieves intrathoracic airway obstruction and reduces cardiac sympathetic activity. Walia et al. [13] are limited by possible systemic bias, as data were collected from the clinical UAS registry database, which did not use standardized BP measurement tools. For future direction, care should be taken to select only data that was collected using standardized methods [5].

Limitations of the Included Studies

Non-randomized designs introduce selection bias. Inconsistent follow-up periods limit comparability. Small sample sizes increase the risk of type II error. Baseline characteristics (age, BMI, gender, race) vary across studies. HNS groups often included CPAP-intolerant patients, creating population bias.

Strengths of This Review

The strengths of this review were as follows: strict inclusion of direct comparative studies, comprehensive outcome evaluation (objective and patient-centered), narrative synthesis appropriate for heterogeneous data, and focus on real-world effectiveness (MDA, adherence, PROs).

## Conclusions

In conclusion, the change in pre- and post-treatment AHI between the HNS and CPAP groups is likely comparable. Both treatments provided a statistically significant decrease in AHI compared to baseline. The current body of evidence demonstrates methodological variability, including differences in sampling strategies and population selection, which may influence the robustness and generalizability of findings. Nevertheless, the available evidence provides valuable insight into the relationship between treatment modalities and AHI outcomes. Findings suggest that greater MDA in the HNS group compared with CPAP may indicate improved treatment adherence or greater reductions in AHI. A significant improvement in ESS and PROs in the HNS group is supported by various studies, which suggest that HNS is more effective than CPAP in improving quality of life and reducing daytime sleepiness. Thus, this supports the hypothesis that HNS has a greater overall efficacy than CPAP; however, based on the limitations of the studies discussed, we cannot conclude whether HNS is a more effective treatment for OSA compared to CPAP. HNS has the potential to serve as an effective alternative to treat OSA for those intolerant to CPAP. Future studies should focus on primary measures (AHI), secondary measures (PROs), and MDA and increase their sample size to draw a definitive conclusion on the relative efficacy between HNS and CPAP.

## Appendices

Appendix A 

**Table 5:** Equations for variables of interest. Depiction of equations used to calculate apnea-hypopnea index (AHI), mean disease alleviation (MDA), objective compliance, and therapeutic efficiency. Adapted from [9].

Variable	Calculation components
Effectiveness of treatment AHI	(Treatment AHI * % Adherence to Therapy) + (Non-Treatment AHI * % Non-Adherence to Therapy)
MDA	Objective Compliance * Therapeutic Efficiency
Objective compliance	[(Objective use of Device [hours per day]) / TotalSleepTime (estimated 7 hours)] * 100
Therapeutic efficiency	AHI (baseline) – AHI (after intervention)
